# Ball back in Africa's court: funding malaria control and elimination

**DOI:** 10.11604/pamj.2013.14.78.2417

**Published:** 2013-02-26

**Authors:** Sylla Thiam, Victoria Kimotho, Teguest Guerma, Jane Carter

**Affiliations:** 1 African and Medical Research Foundation, Nairobi, Kenya

**Keywords:** Malaria, funding, control and elimination, endemic

## To the editors of the Pan African Medical Journal

The success of malaria control and elimination programmes in African countries is being showcased as an example of effective donor support and the benefits of global investment in health. The unprecedented increase in funding from US$ 200 million in 2004 to US$ 1.8 billion in 2010 has resulted in a 50% reduction of malaria cases in 11 African countries and 1.1 million malaria deaths in children averted between 2001 and 2010 [1]. The main donors are the Global Fund against AIDS, Tuberculosis and Malaria (GFATM), the United States Agency for International Development through the President&apos;s Malaria Initiative (PMI), and the World Bank [2]. In countries where effective malaria interventions have been scaled up, child mortality has fallen more than 8% per year, enough to halve child mortality in about a decade [3].

With the current financial crisis, future commitments are uncertain especially from the GFATM, the main donor for malaria [4], whose current contributions range from 22-223% of governments&apos; health expenditures in 12 African countries [5]. Countries remain at different stages of malaria control: some have not yet scaled up interventions; others are in the process of sustaining universal coverage and reducing transmission; some are moving towards or are on track for malaria elimination [1]. Reduced levels of financial support are likely to have a significant impact on health expenditures in at least 15 countries in Africa [5], and represent a major threat for malaria control and elimination. Can Africa find home-based solutions to sustain these successes and take them forward?

Approximately US$ 5-6 billion are needed annually to increase and sustain universal coverage of malaria interventions, reduce malaria deaths to zero and save about 3 million lives by 2015 [1]. If progress is not sustained, a resurgence of the disease could be fatal for the large numbers of people who have lost their natural immunity to malaria from reduced transmission over the last decade. Under the leadership of the World Health Organization (WHO), countries are in the process of developing strategies to address new challenges in malaria control focusing on insecticides and drug resistance. The RTS,S/AS01 vaccine that can reduce malaria risk among children [6] also shows potential, but requires huge resources for implementation, and countries need to plan for it now.

This funding crisis represents a window of opportunity for malaria endemic countries in Africa to invest more in health over the next decade and make their own contributions towards healthy populations. Government funding for malaria in Africa is generally less than US$ 1 per person at risk which represents a very small proportion of the total financing required in the most highly endemic countries [1]; but with ready availability of external funding in past years, domestic funds have not in fact been fully utilised. Since donor funding is unequally distributed and often not tailored to country needs [7, 8], this is the right time for governments to look internally to increase their own funding support and plan their own health agendas.

Many countries have the potential to expand domestic financing for HIV/AIDS, tuberculosis and malaria programmes [5]. Governments need to set up mechanisms within their national budgets for optimal management of resources and explore ways of making drugs and commodities more accessible and affordable. This can best be achieved by promoting community initiatives and better management of local resources such as the Bamako Initiative [9]. In addition, community-based health insurance schemes that facilitate access to treatment and reduce pressure on governments and households need to be further explored and implemented [10].

Africa has real potential to support its own development and all Africans globally need to contribute to this objective. The time has come for the African continent to invest in its own internal capabilities and mobilize its own resources, and move away from reliance on external donor aid.
